# Chronic myeloid leukemia extramedullary blast crisis presenting as central nervous system leukemia: A case report

**DOI:** 10.1097/MD.0000000000013131

**Published:** 2018-11-09

**Authors:** Mingwei Jin, Chengmin Xuan, Jizhao Gao, Rui Han, Shumei Xu, Lei Wang, Yuan Wang, Kunpeng Shi, Sunil Rauniyar, Qi An

**Affiliations:** a Department of Hematology, Xuzhou Children's Hospital, Xuzhou; b Department of Hematology, Affiliated Hospital of Xuzhou Medical University; c The Graduate School, Xuzhou Medical University, Xuzhou, Jiangsu, China.

**Keywords:** *BCR/ABL1*, childhood chronic myeloid leukemia, extramedullary blast crisis, imatinib, Philadelphia (Ph) chromosome

## Abstract

**Rationale:**

Childhood chronic myeloid leukemia (CCML) is a malignant disease of granulocyte abnormal hyperplasia that is caused by clonal proliferation of pluripotent stem cells. The condition is relatively rare, accounting for 2.0% to 3.0% of cases of childhood leukemia. In addition, the incidence of extramedullary blast crisis in CCML presenting as central nervous system (CNS) blast crisis remaining chronic phase of the disease in bone marrow is extremely unusual.

**Patient concerns:**

We report a case of childhood chronic myelogenous leukemia that abandoned treatment, resulting in chronic myelogenous leukemia transforming into extramedullary blast crisis resulting in CNS leukemia, accompanied by the chronic phase of the disease in bone marrow.

**Diagnoses:**

Chronic myeloid leukemia extramedullary blast crisis presenting as CNS leukemia without blast crisis in bone marrow.

**Interventions:**

Following high-dose systemic and intrathecal chemotherapy, the patient continued to do well.

**Lessons:**

High-dose systemic and intrathecal chemotherapy is safe and helpful for CCML extramedullary blast crisis. A long-term follow-up is crucial.

## 1 Introduction

The majority of cases of childhood chronic myelogenous leukemia cases are characterized by the expression of the breakpoint cluster region/Abelson (*BCR/ABL*) fusion gene, which is a product of the Philadelphia (Ph) chromosome. A Ph chromosome is a derivative of chromosome 22 that is formed by a reciprocal translocation between the chromosome 22 long arm and chromosome 9.^[1,2]^ The presence of a Ph chromosome is visible in most chronic myeloid leukemia cells, some cells in acute lymphoblastic leukemia, and a small number of cells in acute myeloid leukemia.^[3,4]^ Three clinically important variants encoded by the Ph chromosome are the p190, p210, and p230 isoforms,^[5]^ where p210 is closely associated with childhood chronic myeloid leukemia (CCML) and can abnormally increase the activity of tyrosine protein kinase, thus causing an increase in the phosphorylation level of downstream proteins, resulting in cells becoming malignant.^[6]^ Therefore, p210 is the primary cause of childhood chronic myelogenous leukemia and drugs targeting p210 have become the top research priority in the treatment of childhood chronic myelogenous leukemia.

In this report, we describe the case of a 5-year-old child with CML with extramedullary blast crisis in the central nervous system (CNS) without blast crisis in bone marrow.

## 2 Case report

**Figure 1:**
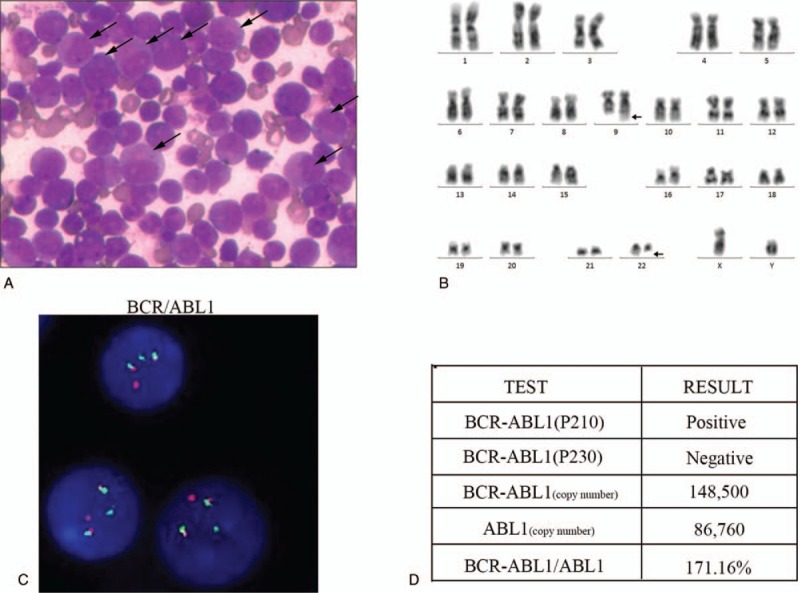
Examination for the primary diagnosis. (A) A large accumulation of leukemic cells in the bone marrow. (B) A G-banded karyotype of bone marrow analysis showing a Philadelphia chromosome. (C) The fusion of *BCR/ABL1* loci was detected by fluorescence in situ hybridization using the Vysis Extra Signal probeyielding red-green fusion signal. (D) Detection of *BCR/ABL1* fusion gene copy number by real-time quantitative polymerase chain reaction.

**Figure 2:**
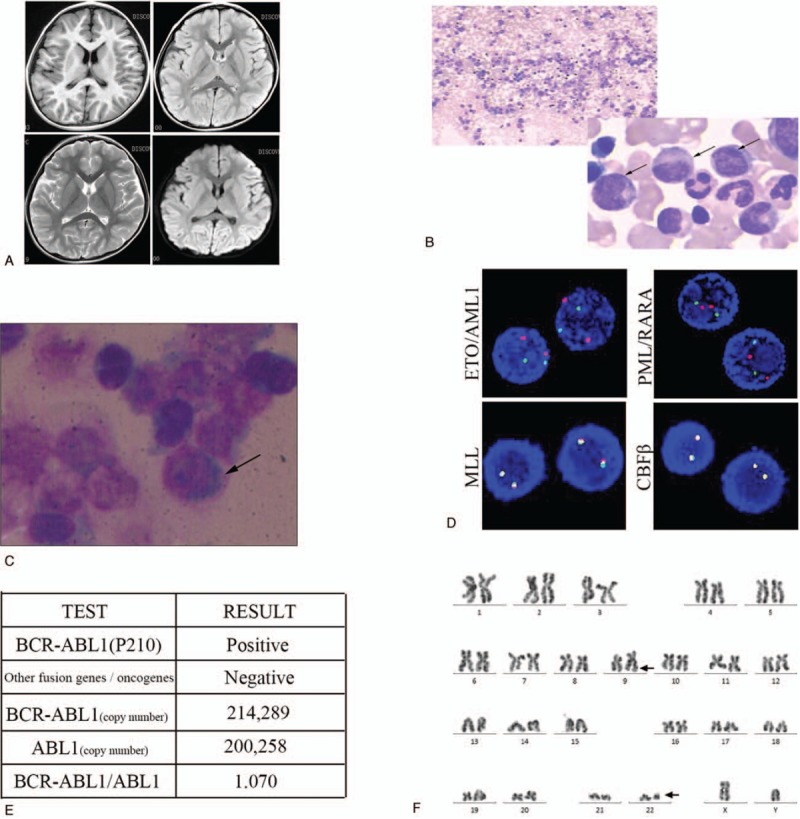
Examination after hospitalization 8 months later. (A) There were no obvious abnormalities on the head magnetic resonance imaging. (B) Bone marrow aspiration reveals large amounts of middle and late granulocytes. (C) Granulocytes are found in cerebrospinal fluid depositions. (D) Fluorescence in situ hybridization shows no abnormalities in the *ETO/AML1*, *MLL*, *PML/RARA*, or *CBFβ* loci. (E) Detection of the copy number of the major type *BCR/ABL1* fusion gene by real-time quantitative polymerase chain reaction. (F) Karyotype analysis showing a Philadelphia chromosome.

A 5-year-old boy with spleen enlargement as the initial presentation was admitted to our hospital in July 2016 and diagnosed with chronic myelogenous leukemia without family-related genetic history (Fig. 1 A–D). After the diagnosis of the disease, the family members gave up treatment and regular re-examination was recommended, requiring discharge. Eight months later, the patient was admitted to our hospital with a headache but no fever or weight loss. There was no obvious abnormality on brain magnetic resonance imaging examination (Fig. 2A). Ultrasonography revealed multiple enlarged lymph nodes in the neck, armpits, groin, and umbilicus, and the liver and spleen were slightly enlarged.

Initial examination of peripheral blood counts showed white blood cells 19.99 × 10^9^/L (normal: 4–10 × 10^9^/L), hemoglobin 130 g/L (normal: 110–160 g/L), platelets 273 × 10^9^/L (normal: 100–300 × 10^9^/L). Bone marrow aspirate smears revealed active hyperplasia of bone marrow, and the ratio of G/E (granulocytes to erythrocytes) was 12.91:1. The proportion of granulocyte was increased, accounting for 77.5%, and the proportion of neutrophils and the following stages of cells were increased (Fig. 2B).

Bone marrow immunophenotyping found that lymphocytes accounted for around 7.5% of the nucleated cells, a significantly reduced proportion. The original region cells accounted for about 0.5% of the nucleated cells and were scattered. Mononuclear cells accounted for about 3.5% of the nucleated cells, indicating phenotypic maturation. Granulocytes accounted for about 85.5% of the nucleated cells, a significantly higher proportion. Cerebrospinal fluid immunophenotyping showed that the original cell distribution area contained abnormal cell populations, accounting for about 85.5% of nucleated cells, with expressions of HLA-DR, CD13, CD19, CD33, CD34, CD38, CD58, CD117, CD123, TdT, and partial expression of CD10 (Fig. 2C). No abnormal signals of *ETO/AML1*, *MLL*, *PML/RARA*, and *CBFβ* detection sites were detected by fluorescence in situ hybridization (Fig. 2D). Fusion gene detection showed that the *BCR/ABL1* (p210) fusion gene was positive and with high copy rate (Fig. 2E). A chromosome karyotype analysis showed 46, X, Y, t (9;22) (q34/q11) (3)/46, XY (2) (Fig. 2F).

The child was diagnosed with chronic myeloid leukemia extramedullary blast crisis presenting as CNS leukemia without blast crisis in bone marrow. Extramedullary blast crisis of CML is defined by infiltration of leukemic blasts in areas other than bone marrow, which has been reported in only 4% to 16% of CML cases during the disease Course.^[7]^

## 3 Results

**Figure 3:**
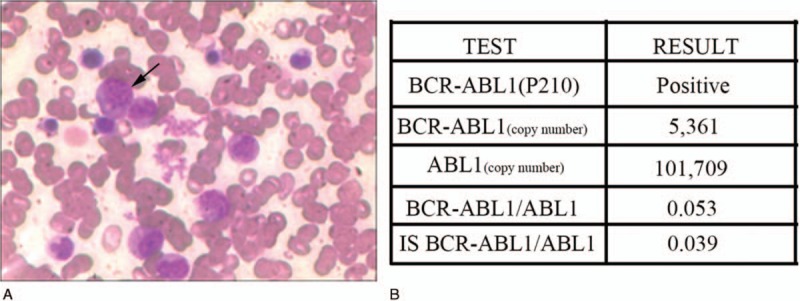
Examination after treatment. (A) Bone marrow smears show a significant decrease in abnormal granulocytes. (B) Detection of the copy number of the major type *BCR/ABL1* fusion gene by real-time quantitative polymerase chain reaction.

He was given methotrexate (12.5 mg), dexamethasone (5 mg), and cytarabine (35 mg) intrathecal injection therapy. The program of intrathecal injection treatment was 3 times the 1st week, twice during the 2nd and 3rd weeks, and once during the 4th week. He was also given the HAD chemotherapy regimen (homoharringtonine 3 mg/m^2^·d for 1–7 days, cytarabine 200 mg/m^2^·d divided into 2 injections for 1–7 days, and daunorubicin 40 mg/m^2^·d for 1–3 days). The HAD chemotherapy regimen was carried out 3 times, and he continued to receive the 2nd-generation tyrosine kinase inhibitors (TKIs) treatment. The patient refused the treatment recommendation of allogeneic hematopoietic stem-cell transplantation (allo-HSCT) and cranial and spinal radiation therapy due to economic reasons. After the treatment, the enlarged nodes and cerebrospinal fluid were completely normal and his bone marrow and *BCR/ABL1* copy number were re-examined (Fig. 3A, B). Following high-dose systemic and intrathecal chemotherapy, the patient continued to do well in the last review (11 October 2018).

## 4 Discussion

Because of the low incidence of pediatric chronic myelogenous leukemia, there are relatively few reports about it.^[8]^ We reported a case of chronic myelogenous leukemia extramedullary blast crisis with CNS leukemia. Below, we present a brief discussion of the pathogenesis of chronic myelogenous leukemia, acute changes, and CNS leukemia. Despite the increasing accuracy of diagnosis and effectiveness of treatment, there are still cases of chronic myelogenous leukemia that do not respond and undergo acute changes.

Recent studies have found that blast crisis in chronic myelogenous leukemia is closely related to chromosomal abnormalities and genetic mutations.^[9–11]^ In addition to *BCR/ABL1* fusions, there are mutations in *p53*,^[12,13]^*RUNX1*,^[14,15]^*RAS*,^[16]^ and other genes during a CCML blast crisis. Gene mutations are associated with acute changes and are also related to imatinib resistance.^[17]^

Imatinib, a specific TKI, is currently the 1st-line drug in the treatment of chronic myeloid leukemia.^[18]^ For drug-resistant patients, the dose can be increased or a 2nd-generation targeted BCR/ABL1 tyrosine kinase inhibitor, such as nilotinib, can be used.^[19]^ Although the 2nd-generation TKIs (nilotinib and dasatinib) have an improved penetration of the blood–brain barrier, single cases of isolated CNS blast crises have also been reported.^[20]^ So far, allogeneic hematopoietic stem-cell transplantation is still the only way to cure chronic myelogenous leukemia and is one of the 1st treatment options for children with chronic myelogenous leukemia.^[21]^

At present, the pathogenesis of CNS leukemia is not clear. Leukemia cells may enter the CNS through blood source diffusion, meningeal implantation, cranial bone marrow infiltration, or lumbar puncture injury.^[22]^ The treatment of CNS leukemia mainly includes methotrexate, cytarabine, dexamethasone, triple intrathecal chemotherapy, and systemic chemotherapy. A previous case reported complete remission of patients with chronic myeloid leukemia relapse presenting with CNS blast crisis and bilateral optic nerve infiltration after systemic, intrathecal chemotherapy, radiation, and allogeneic hematopoietic stem-cell transplantation.^[23]^ Although our patients received a significant response, we acknowledge that leukemia cells in the space surrounding the nervous system are not completely eliminated. We recommend radiation therapy and allogeneic hematopoietic stem-cell transplantation. However, due to economic reasons, the parents refused further treatment. Although the efficacy of medical treatment has been greatly improved, CNS leukemia remains the major cause of leukemia relapse and treatment difficulties.

We reported a case of childhood chronic myelogenous leukemia in which a child experienced an extramedullary acute crisis presenting as CNS leukemia because of abandoning treatment. After active treatment, the patient's symptoms were relieved. It is necessary to conduct further study into the relevant mechanisms of this disease to provide a basis for its treatment.

## Acknowledgment

The authors thank Daihua Fang of Xuzhou Children's Hospital Bone marrow Laboratory and Beijing Hester clinical inspection institute for the relevant pictures.

## Author contributions

**Data curation:** Mingwei Jin, Chengmin Xuan, Jizhao Gao, Rui Han.

**Funding acquisition:** Qi An.

**Investigation:** Shumei Xu, Lei Wang.

**Resources:** Yuan Wang.

**Software:** Kunpeng Shi, Shumei Xu.

**Writing – original draft:** Mingwei Jin, Kunpeng Shi.

**Writing – review & editing:** Chengmin Xuan, Sunil Rauniyar, Qi An.
